# Methods to Evaluate the Effect of Ethanol on the Folate Analogue: Fluorescein Methotrexate Uptake in Human Proximal Tubular Cells

**DOI:** 10.1155/2009/291349

**Published:** 2009-09-29

**Authors:** Sivakumar JT Gowder, Kenneth E. McMartin

**Affiliations:** ^1^Department of Pharmacology, LSU Health Sciences Center, Shreveport, LA 71118, USA; ^2^Trinity University School of Medicine, Kingstown, St. Vincent, P.O. Box 2394, West Indies

## Abstract

Ethanol-induced folate deficiency is due to effects of ethanol on folate metabolism and absorption. We have already shown by using different methods that ethanol interferes with reabsorption of folate from the proximal tubule. In this study, we have used the folate analogue, the fluorescein methotrexate (FL-MTX), in order to evaluate effects of ethanol on FL-MTX uptake by the human proximal tubular (HPT) cells by using a confocal microscope and fluoroskan microplate reader. Since endothelins (ETs) play a major role in a number of diseases and also in the damage induced by a variety of chemicals, we have used endothelin-B (ET-B) and protein kinase-C (PKC) inhibitors to evaluate the role of endothelin in ethanol-mediated FL-MTX uptake by using fluoroskan microplate reader. Confocal microscope and fluoroskan studies reveal that cellular absorption of FL-MTX is concentration-dependent. Moreover, ethanol concentration has an impact on FL-MTX uptake. Fluoroskan studies reveal that the ethanol-induced decrease in FL-MTX uptake is reversed by adding the ET-B receptor antagonist (RES-701-1) or PKC-selective inhibitor (BIM). Thus, we can conclude that ethanol may act via ET and ET in turn may act via ET-B receptor and the PKC signaling pathway to impair FL-MTX transport.

## 1. Introduction

Folate is a water-soluble vitamin that occurs in natural food sources as reduced methylated or formylated tetrahydrofolate. Folic acid is a synthetic analogue with no specialized metabolic activity [1]. Folate is essential for cellular proliferation and tissue regeneration. As mammalian cells cannot synthesize folates de novo, tightly regulated cellular uptake processes have evolved to sustain sufficient levels of intracellular tetrahydrofolate cofactors to support biosynthesis of purines, pyrimidines, and some amino acids [2]. Folate has emerged as a key nutrient for optimizing health and impaired folate status has been found as a risk factor for cancer, cardiovascular disease, and neurocognitive disorders [3]. Plasma folate seems to be a suitable marker for assessment of folate status for use in large-scale epidemiological studies [4]. 

Renal tubular reabsorption of filtered folate is essential for the conservation and normal homeostasis of this important vitamin [5]. Although reabsorption across the apical (AP) membrane of the renal proximal tubule cell plays a vital role in the conservation of plasma 5-methyltetrahydrofolate, basolateral (BL) membrane-directed secretory pathways may also be important in regulating the urinary excretion of folate [6]. The reduced folate carrier (RFC) plays a critical role in the cellular uptake of folates. However, a little is known regarding the mechanism used to transport substrates or the tertiary structure of the protein [7].

Epidemiologic studies on humans and experimental studies on animals show that alcohol causes cancer through different mechanisms, including through mutagenesis by acetaldehyde, through perturbation of estrogen metabolism and response, and through inducing oxidative damage and/or by affecting folate and one-carbon metabolism pathways [8]. Ethanol-induced folate deficiency is due to the effects of ethanol on folate metabolism and absorption. We have already shown by using different methods that ethanol interferes with the reabsorption of folate from the proximal tubule. Acute ethanol ingestion by human alcoholic subjects produces a marked decrease in serum folate levels in 16 hours [9]. In rats, acute doses of ethanol produce a marked increase in urinary folate excretion which precedes a decrease in plasma folate levels [10]. Studies on acute ethanol in cultured human proximal tubular (HPT) cells have shown the inhibition of reabsorptive transport of 5-methyltetrahydrofolate [11]. 

In this study, we used the folate analogue, the fluorescein methotrexate (FL-MTX), as a model compound of our study to evaluate the effect of ethanol on FL-MTX uptake by the HPT cells by using confocal microscope and fluoroskan microplate reader. Fluorescein methotrexate (F-MTX), a fluorescent derivative of MTX, was synthesized by coupling the carboxyl of the glutamate moiety of MTX through a diaminopentane spacer to fluorescein isothiocyanate [12]. We used our HPT cells to elucidate the mechanism of FL-MTX since these cells provide a good model to understand the mechanism of transport studies. Endothelins (ETs) are polypeptide hormones that are potent vasoconstrictors [13]. In the kidney, endothelin plays a major role in controlling water and sodium excretion and acid base balance and it is participated in acute and chronic renal failure [14]. Since ETs play a major role in a number of diseases and also in the damage induced by a variety of chemicals [15], we have used Endothelin-B (ET-B) and protein kinase-C (PKC) inhibitors to evaluate the role of endothelin in ethanol-mediated FL-MTX uptake by using fluoroskan microplate reader.

## 2. Materials and Methods

### 2.1. Materials

FL-MTX, BIM (Bisindolylmaleimide I, PKC- Selective Inhibitor) were purchased from Molecular Probes (Eugene, Ore, USA) and RES-701-1 (ET-B receptor antagonist) was purchased from American Peptide Company (Sunnyvale, Calif, USA). We purchased all other chemicals used in this study from Sigma Chemical Co (St. Louis, Mo, USA), Collaborative Biomedical Research (Bedford, Mass, USA) and GIBCO (Grand Island, NY, USA). 

### 2.2. Cell Culture

HPT cells from normal human kidney cortex tissue (from kidneys unable to be used in transplantation) were isolated by the enzyme dissociation method using a DNAase-collagenase mixture as described previously [16]. Isolated cells were regularly cultured on collagen-coated 75 cm^2^ flasks (Costar, Cambridge, Mass, USA) in a serum-free mixture of Dulbecco's modified Eagle's medium-Ham's F-12 medium (50 : 50 by volume) with the following additions per L: selenium (5 *μ*g), insulin (5 mg), transferrin (5 *μ*g), hydrocortisone (36 *μ*g), epidermal growth factor (10 *μ*g), triiodothyronine (4 ng), and 2 mmol glutamine. When the HPT cells were grown to confluency in tissue culture flasks, the cells were subcultured onto 24-well plates (Costar, Cambridge, Mass, USA). We used the cells when they attained confluency for this study.

### 2.3. Confocal Microscopy

HPT cells in the 24 well plates were washed twice with room temperature buffer (PBS). The cells were treated with (a) 1.5 mL of PBS with 0.5,1,2, and 4 *μ*M of FL-MTX and (b) 4 *μ*M of FL-MTX and different concentrations (0,500 and 750 mg/dL) of ethanol for 30–45 minutes of incubation at 37°C. The cells were subjected to confocal microscopy.

The cells in the 24 well plates were directly observed under Bio Rad Radiance 2000 Confocal microscope and viewed through a 40X CF infinity plan fluor objective. 

Excitation was provided by the 488 nm line of an argon laser. A 515/30 nm filter and a 545/40 nm dichroic filter were used. The trapped signal was sent inside the Photo Multiplier Tubes which capture light using total internal reflection. A few cells in the wells were selected and the images were viewed. Bio-Rad's Lasersharp-I allows 32-bit image acquisition, display and 3D volume rendering, and image analysis.

### 2.4. Fluoroskan Microplate Reader

HPT cells in the 24 well plates were washed twice with room temperature pH 7.4 incubation buffer (20 mM HEPES, pH 7.4, containing NaCl, KCl, CaCl_2_, MgCl_2_, D-glucose, and NaHCO_3_) and then treated with (a) 1.5 mL of the above incubation buffer containing 1, 2, 4, 8, and 16 *μ*M FL-MTX, (b) 1.5 mL of incubation buffer containing 10 *μ*M FL-MTX and different concentrations (0, 100, 200, 300, 400, and 500 mg/dL) of ethanol, and (c) 1.5 mL of incubation buffer containing 10 *μ*M FL-MTX, 500 mg/dL ethanol and 10 *μ*M ET-B receptor antagonist (RES-701-1) or 2.5 *μ*M PKC selective inhibitor (BIM), respectively, for 2 hours of incubation at 37°C. The cells were washed with incubation buffer and then the fluorescence intensity was read in fluoroskan reader with an appropriate filter.

### 2.5. Statistical Analysis

Data are expressed as mean ± SEM (*n* = 5). All statistical analysis was carried out using SAS 9.1 (SAS Institute, Cary, NC, USA). One way analysis of variance with Duncan's multiple range tests was performed to identify the statistical significance. A *P*- value of less than .05 was considered statistically significant.

## 3. Results

**Figure 1:**
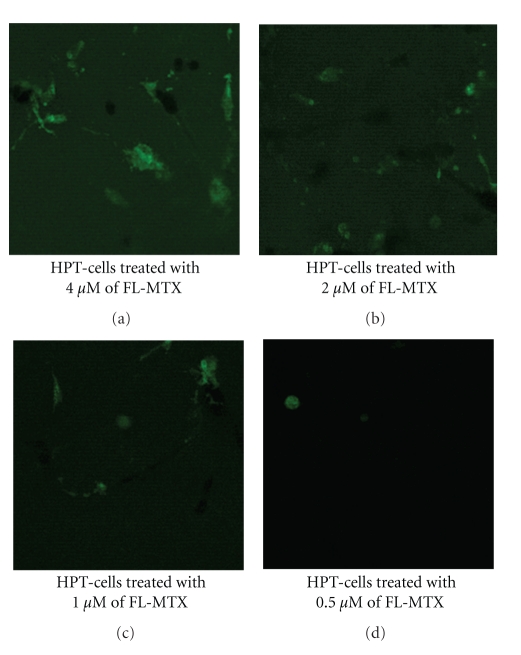
Confocal images of FL-MTX uptake by HPT cells in 24 well plates. Cellular fluorescence was more when the cells treated with 4 *μ*M FL-MTX and it was gradually decreasing when the cells were treated with 2 *μ*M, 1 *μ*M, and 0.5 *μ*M FL-MTX. Thus, FL-MTX uptake was directly proportional to its concentrations.

**Figure 2:**
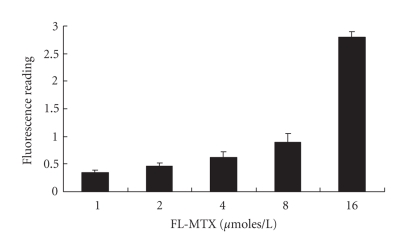
Fluoroskan studies of FL-MTX (10 *μ*M) uptake by HPT cells in 24 well plates. FL-MTX uptake is directly proportional to its concentrations. Data are expressed as mean ± SEM (*n* = 5).

Confocal microscopic (Figure 1) and fluoroskan studies (Figure 2) revealed that cellular absorption of FL-MTX was concentration-dependent. The cellular absorption of different concentrations of FL-MTX uptake is significantly different (*P* < .001) (Figure 2).The cellular fluorescence was more in the cells treated with 4 *μ*M of FL-MTX when compared with the cells treated with less concentrations of FL-MTX (Figure 1). When there was an increase of FL-MTX concentration, there was an increase of FL-MTX uptake. Thus, the uptake of FL-MTX was found to depend on its extracellular concentration and conforms to Michaelis- Menten kinetics. That is, initial velocities of transport of FL-MTX increased in proportion to its concentrations. 

**Figure 3:**
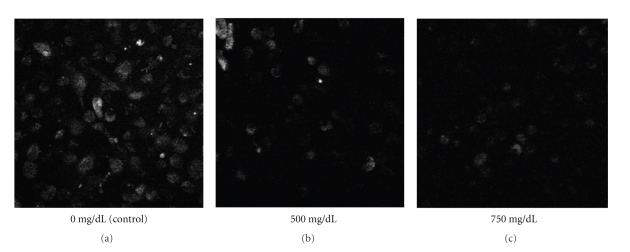
Confocal images of HPT cells in 24 well plates treated with FL-MTX (4 *μ*M) and different concentrations of ethanol (0, 500, and 750 mg/dL). The images reveal that ethanol concentration has an impact on FL-MTX uptake.

**Figure 4:**
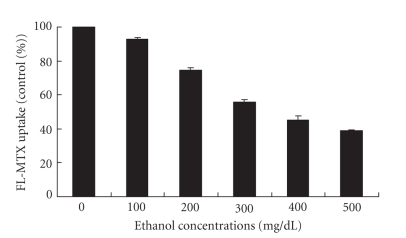
Fluoroskan studies of FL-MTX (10 *μ*M) uptake by HPT cells in 24 well plates treated with different concentrations (0, 100, 200, 300, 400, and 500 mg/dL) of ethanol. Ethanol concentration has an impact on FL-MTX uptake. Data are expressed as mean ± SEM (*n* = 5).

Moreover, ethanol concentration was found to have an impact on FL-MTX uptake. When there was an increase in ethanol concentration, there was a decrease in FL-MTX uptake (Figures 3 and 4). For confocal studies, we used 0, 500, and 750 mg/dL ethanol. From the confocal images, we can observe that the cellular fluorescence of FL-MTX of the cells treated with ethanol at the concentration of 500 mg/dL was less when compared with controls and more when compared with the cells treated with 750 mg/dL (Figure 3). For fluoroskan studies, we used various concentrations of ethanol 0, 100, 200, 300, 400, and 500 mg/dL and observed that the FL-MTX uptake was gradually decreased when the concentrations of ethanol were increased and the effect of different concentrations of ethanol on cellular uptake of FL-MTX is significantly different (*P* < .001), (see Figure 4). We used 4 *μ*M and 10 *μ*M FL-MTX for confocal and fluoroskan studies when used with different concentrations of ethanol. We standardized these FL-MTX concentrations based on different FL-MTX uptake experiments by using confocal microscopy and fluoroskan microplate reader. 

**Figure 5:**
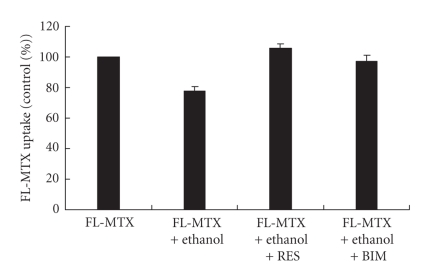
Fluoroskan studies of FL-MTX (10 *μ*M) uptake by HPT cells in 24 well plates treated with ethanol (500 mg/dL), ET-B receptor antagonist (RES-701-1) (10 *μ*M), and PKC selective inhibitor (BIM) (2.5 *μ*M). Inhibition of FL-MTX uptake is reversed by the addition of RES-701-1 or BIM. Data are expressed as mean ± SEM (*n* = 5).

Fluoroskan studies revealed that ethanol-induced decrease in FL-MTX uptake was reversed and it is significant (*P* < .001) by adding ET-B receptor antagonist (RES-701-1) or PKC selective inhibitor (BIM) (Figure 5). We standardized from different experiments and hence we used 500 mg/dL ethanol, 10 *μ*M FL-MTX, 10 *μ*M ET-B receptor antagonist (RES-701-1), or 2.5 *μ*M PKC-selective inhibitor (BIM) for this study. From this experiment, we can suggest that ethanol may act via ET and ET in turn may act via ET-B receptor and PKC signaling pathway to impair FL-MTX transport. We standardized the concentrations of RES-710-1 and BIM in this study based on various experiments.

## 4. Discussion

For folate uptake and other toxicological studies, we are traditionally growing HPT cells, which formed a useful tool to understand various mechanisms. HPT cells which were fully grown and confluent in 24 well plates were used for this study. The cells form confluent monolayers on plastic as well as on porous membrane inserts and produce domes (hemicysts) in culture were indicating retention of ion transport properties and integrity of cell to cell junctions. Before conducting the present experiment, we also characterized these cells histochemically by using our recently developed methods for gamma glutamyl transpeptidase (GGT) and glucose-6-phosphatase (G-6-P) (data not shown). The cells were positive for GGT and G-6-P. The cells, thus, retained the membrane integrity and function like that of the intact proximal tubule and hence we used these cells to develop confocal and fluoroskan methods to understand the mechanism of FL-MTX uptake studies. 

We developed our confocal method in such a way so as to demonstrate the images in 24 well plates directly. When we treated the HPT cells with different concentrations of FL-MTX and viewed by using the confocal microscope, we observed that cellular uptake of FL-MTX is concentration-dependant. Fluoroskan method also showed that cellular uptake of FL-MTX was concentration dependant. In general, transport on the “classical” organic anion system in renal proximal tubule is specific and active. Assaraf et al. [17] demonstrated that FL-MTX is accumulated in cells by a passive diffusion process. Miller [18] realized that the secretion of large organic anion, that is, FL-MTX is handled by a separate and distinct organic anion transport system. Masereeuw et al. [19] suggested that cellular accumulation of FL-MTX is a result of diffuse entry and it is energy-independent. FL-MTX transport by RFC in human leukemia cell lines and leukemia blasts was demonstrated by confocal laser scanning microscopy [20]. Li et al. [21] observed that the transport of FL-MTX is mediated by the reduced folate transporter (RFC) in brush border membrane vesicles of rat small intestine. The reduced folate carrier (RFC), a facilitative transporter, plays a major role in the delivery of reduced folates and antifolates into cells [22]. Thus, FL-MTX uptake was regulated by RFC in this experiment. 

 It is quite clear from both the confocal and fluoroskan methods that ethanol reduced FL-MTX uptake in a concentration dependant manner. Since the confocal methods were quite comparable with our fluoroskan methods, we concluded that our protocol was quite clear for both the methods. Miller [15] observed that a few drugs impaired FL-MTX transport with respect to their concentration. Villanueva et al. [23] reveals that chronic ethanol exposure decreases the absorption of folic acid by altering the expression of RFC and consequently its transport kinetics. Our previous studies indicate that ethanol directly impairs the renal conservation of 5-methyltetrahydrofolate [11]. From these studies, we can assess that ethanol impairs FL-MTX and also its analogues, 5 M and folic acid.

Chronic ethanol decreases endothelin-stimulated glucose uptake in rat adipocytes [24]. Folate supplementation improves endothelial function while in hyperhomocysteinemia [25]. These reports provide ample evidences regarding endothelin function in FL-MTX transport. In order to evaluate the mechanism of ethanol-mediated FL-MTX uptake studies, we used ET-B receptor antagonist (RES-701-1) or PKC-selective inhibitor (BIM). The experiments on these compounds show that ethanol may act via ET and ET in turn may act via ET-B receptor and PKC signaling pathway to impair FL-MTX uptake. Renal ET production is associated with a number of renal diseases and also with the action of chemical toxicants [15]. Urinary ET-1 excretion increases in renal failure from a variety of causes including radio contrast nephropathy and chemical therapy [26, 27]. The toxic chemicals normally alter tubular function through ET receptor [28]. The effect of ethanol on FL-MTX transport was abolished by adding ET-B receptor antagonist, RES-701-1, or by adding PKC-selective inhibitor-BIM to the HPT cells in 24 well plates in this study. Endothelin-B exerts its effect through Protein Kinase C [29, 30]. Hamid and Kaur [31] revealed the role of PKC in intestinal folate transport during alcohol conditions. Although blocking any of the steps in the ET-ETB-NOS-NO-PKC pathway eliminates the effect of certain chemicals or nephrotoxicants on transport pathway, just blocking ETB receptor, the first step in signaling is sufficient [15]. We used both ET-B receptor antagonist and PKC inhibitor in different times in order to limit the effect of ethanol on transport. 

By using the folate analogue, FL-MTX, we developed this method to evaluate the mechanism of ethanol mediated FL-MTX transport. Our uptake methods by using confocal microscope and fluoroskan microplate reader showed that ethanol-impaired FL-MTX uptake was mediated through endothelin signaling. Our HPT cells in 24 well plates provided a good model for this study. On the therapeutic point of view, ET receptor antagonists may prevent ethanol impaired FL-MTX or folate uptake.
